# Advancing Knowledge, Improving Practice: NIAAA Research Priorities and Funding Opportunities

**Published:** 1995

The National Institute on Alcohol Abuse and Alcoholism (NIAAA) of the National Institutes of Health (NIH) is the primary Federal entity responsible for research on the causes, consequences, treatment, and prevention of alcohol-related problems. NIAAA conducts and supports biomedical and behavioral research into the effects of alcohol on the human mind and body, prevention and treatment of alcohol abuse and alcoholism, and epidemiology of alcoholism and alcohol-related problems.

Research results are presented at conferences and featured in a wide range of publications, including scientific journals, brochures specifically written for a lay audience, and NIAAA’s World-Wide Web site. To ensure rapid dissemination of alcohol research findings, NIAAA produces the Alcohol and Alcohol Problems Science Database (ETOH), a bibliographic database of more than 90,000 records of alcohol research findings. NIAAA also maintains the Alcohol Epidemiologic Data System, which acquires, analyzes, and provides epidemiological statistics on alcohol-related subjects, and the computer bulletin board “Quick Facts,” an online alcohol statistics database. NIAAA’s publications include a quarterly, peer-reviewed journal, *Alcohol Health & Research World;* reports and monographs, such as the triennial *Special Report to the U.S. Congress on Alcohol and Health;* and the quarterly *Alcohol Alert* bulletin for clinicians and other health professionals. To further promote dissemination of ongoing research, NIAAA convenes several conferences and workshops each year.

## Twenty-five Years of Research Progress

During NIAAA’s 25-year history, researchers have made much progress in understanding the causes, consequences, prevention, and treatment of alcohol-related problems. Highlights of this progress are described below.

### What is alcoholism and what accounts for it?

The key question in alcoholism research is, Why do some people exhibit a pathological appetite for alcohol? NIAAA has dedicated a large portion of its research to answering this question. Studies using experimental animals have shown that the actions of alcohol that cause intoxication, reinforce drinking behavior, and lead to addiction are based principally in the brain. Imaging techniques, such as magnetic resonance imaging (MRI), positron emission tomography (PET), and electro-physiological studies, are helping scientists understand alcohol’s effects on the brain. By combining newly emerging computer-aided modeling with state-of-the-art imaging techniques, researchers are developing a better understanding of addictive behavior. Such information will help clinicians identify people at risk for developing alcoholism.

### Why are some people more vulnerable to alcohol’s effects?

A benchmark in the history of alcoholism research was the demonstration that for many people, susceptibility to alcoholism is inherited. NIAAA has supported studies of the genetics of alcoholism using a range of techniques, from population-based studies to the latest methods of molecular analysis. For example, adoption and twin studies provided the first evidence of a genetic component in the risk for alcoholism. Because genetics alone does not account for the development of alcoholism, researchers also have investigated environmental factors that may put people at risk for alcohol problems. For example, alcohol research has shown that expectancies, particularly beliefs about the positive effects of alcohol, can predict drinking behavior. Researchers currently are focusing on unraveling the genetic factors involved in the transmission of alcoholism from one generation to the next as well as determining the manner and extent to which genetic and environmental factors interact in the development of alcoholism.

### How does alcohol damage the body?

Research in the past 25 years also has generated an increasing awareness of the medical consequences of alcohol and the mechanisms by which they occur. Some of these consequences are alcoholic cirrhosis; cancer; immune defects; and fetal alcohol syndrome and other alcohol-related birth defects. Other consequences of alcohol use include problems with perception, judgment, muscle coordination, memory, and learning, all of which can lead to alcohol-related trauma, including accidents, traffic crashes, and interpersonal violence. Understanding the mechanisms of alcohol’s effects strengthens efforts to decrease alcohol-related consequences. For example, scientists in the alcohol field were responsible for identifying alcohol as a teratogen (i.e., a cause of birth defects). This research led to the 1981 issuance of the first *Surgeon General’s Advisory on the Use of Alcohol During Pregnancy*.

**Figure:**
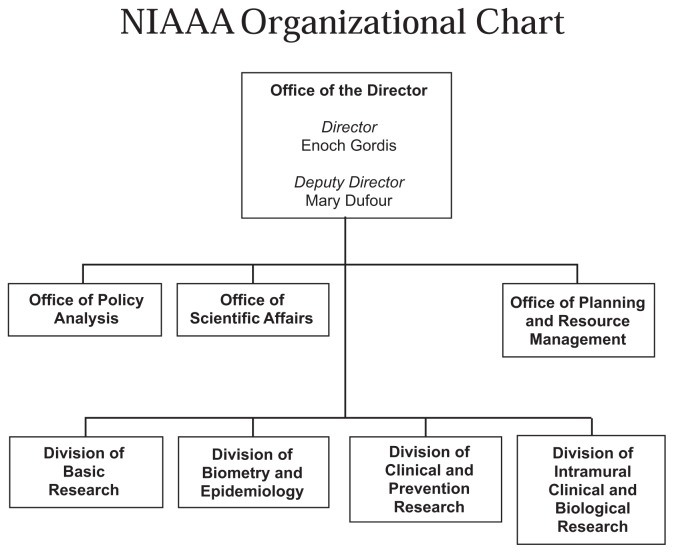


### How can alcohol-related problems be effectively prevented?

Alcohol prevention research includes preintervention and intervention studies. Preintervention studies seek to identify factors that may place people at particular risk as well as factors that may protect them from developing an alcohol problem. Such research leads to models of problem development and behavioral change and allows advance testing of surveys, questionnaires, and other measurement instruments. Intervention studies are designed to develop and test prevention strategies. This research takes advantage of natural experiments (such as implementation of new legislation) to study the effects of public policies on alcohol-related consequences.

Alcohol prevention research provided the scientific basis for the Federal Uniform Drinking Age Act of 1984, which had the effect of raising the minimum legal drinking age to 21. Alcohol-related driving deaths subsequently declined, especially among persons ages 16 to 20. New research is aimed at developing prevention strategies rooted in changing the social, legal, and economic context in which drinking occurs. Innovative tools, such as computer models, are helping scientists analyze the end result of such changes. This type of research can assist policymakers in formulating effective health, education, and economic strategies to reduce the consequences of alcohol misuse.

### How can alcohol-related problems be effectively treated?

During the last decade, modern standards of evaluating treatment outcomes, including the use of controls, blinding, random assignment of subjects, multiple and objective measures of treatment effects, adequate followup to confirm results, and appropriate statistical analysis, have been implemented by alcohol treatment researchers. Controlled clinical trials supported by NIAAA have led to the Food and Drug Administration’s approval for the use of the medication naltrexone as an adjunct to traditional treatment. Another important development in alcohol treatment research is its emphasis on patient-treatment matching. Because no single treatment approach is effective for all people with alcohol problems, NIAAA is sponsoring Project MATCH, a collaborative effort between NIAAA scientific staff and scientists at a number of institutions around the United States, to develop practical guidelines for assigning patients to appropriate treatment based on relevant patient characteristics.

## NIAAA’s Organization

NIAAA is one of 17 research institutes that together create the NIH. To fulfill its research mission, NIAAA supports both intramural (in-house) and extramural research (support for scientists at universities and other institutions throughout the United States). NIAAA’s greatest emphasis is on extramural studies, with approximately 88 percent of the research budget available to extramural researchers. The Office of the Director and three NIAAA staff offices manage and coordinate activities related to extramural science policy and scientific communication; legislation and public policy; collaborative research and international programs; and budget, planning, management, and administration. Research activities are conducted by the Institute’s three extramural research divisions and by the NIAAA Intramural Research Program (IRP) located on the NIH campus in Bethesda, Maryland.

### Staff Offices

- **The *Office of the Director*** initiates and guides the development of Institute policies and programs; coordinates communication between NIAAA and other NIH Institutes, Federal agencies, and the public and professional communities; and manages the Institute’s Equal Employment Opportunity programs. Additionally, the ***Office of Collaborative Research Activities*** within the Office of the Director initiates and conducts collaborative activities with other NIH Institutes, government agencies, and other organizations interested in alcohol-related problems and coordinates and administers the Institute’s international activities and science education programs.
- **The *Office of Policy Analysis*** monitors alcohol-related legislative developments and proposals; conducts and supports studies of alcohol-related policy and economic issues; and provides recommendations for changes in public policies based on research findings.
- **The *Office of Scientific Affairs*** guides the development of the Institute’s broad research policies and programs; administers the peer and objective review of grant applications and contract proposals; develops the policies and rules for the Institute’s Extramural Research Program (ERP); and oversees the Institute’s public information and scientific communication program.
- **The *Office of Planning and Resource Management*** directs the Institute’s administrative support activities, including planning, budget formulation and execution, personnel management, administrative services, and grants and contracts management.

### Research Divisions

- **The *Division of Basic Research*** supports extramural studies in such areas as neuroscience, genetics, and molecular biology to increase understanding of the mechanisms that underlie alcohol addiction and alcohol-related tissue injury.
  The Division also oversees the National Alcohol Research Centers Program, which supports multidisciplinary research around central themes, such as “alcohol and immunology.”
- **The *Division of Clinical and Prevention Research***, another extramural research division, supports studies aimed at developing practical and effective models of treating and preventing alcohol-related problems. Research activities include the development of new medications, preventive interventions designed for specific populations, and treatment protocols that match patients with appropriate treatment elements.
- **The *Division of Biometry and Epidemiology*** supports both extramural and intramural research activities designed to discover the etiology, incidence, and prevalence of alcohol-related problems.
- **The *Division of Intramural Clinical and Biological Research*** administers the Institute’s IRP. The division’s components include four laboratories (molecular and cellular neurobiology, neurogenetics, membrane biochemistry and biophysics, and clinical studies) and a 10-bed alcoholism clinical unit at the NIH Clinical Center.

**NIAAA Research Programs**

**Extramural Research**

NIAAA supports research in a number of broadly defined areas of investigation. Scientists from a variety of disciplines, including biology, medicine, and social and behavioral sciences, participate in the ERP. NIAAA’s categories of research are as follows:

- ***Alcohol and pregnancy***. Investigations of the adverse effects of fetal alcohol exposure on all aspects of fetal growth and development, including development of the brain; the genetic factors contributing to fetal vulnerability to alcohol; and ways to improve identification of and intervention with pregnant women who are at high risk for giving birth to an infant with alcohol-related birth defects.
- ***Alcohol-related medical disorders***. Studies of alcohol’s toxic effects on human organs, including the liver, heart, pancreas, and brain; and alcohol’s effects on the immune system, including alcohol’s role in the acquisition and clinical course of AIDS.
- ***Alcohol-related performance***. Studies of how alcohol affects the ability to perform important tasks; research includes the development of new methods for measuring alcohol impairment.
- ***Biochemistry and metabolism***. Investigations of how the body metabolizes alcohol; how alcohol affects tissue structure and function; and how alcohol alters the metabolism of nutrients, vitamins, and other substances.
- ***Environmental determinants of drinking***. Investigations of the factors in drinkers’ environments that lead to abuse and disease, including legal, social, and economic influences.
- ***Epidemiology and biometry***. Research examining the geographic and demographic distribution (including age, sex, ethnicity, and socioeconomic status) over time of drinking patterns, alcohol abuse and dependence, and alcohol-related injuries, illness, and death.
- ***Genetics and molecular biology***. Research on how genes and the environment interact to precipitate alcoholism, identifying biochemical and physiological markers of high risk and mapping and cloning genes that influence alcohol-related behavior.
- ***Health services research***. Research seeking to develop a knowledge base for improving the efficiency and effectiveness of treatment and prevention services for alcohol-related problems, including studies of how organization, financing, management, and delivery of alcohol-related treatment and prevention services (including managed care) affect service accessibility, quality utilization, cost, and outcome.
- ***Medications development***. Investigations aimed at developing pharmacological interventions to diminish the craving for alcohol, reduce relapse, and safely detoxify dependent individuals undergoing treatment. Studies range from basic biomedical research to clinical applications for the treatment of alcoholism.
- ***Moderate alcohol consumption***. Molecular, cellular, epidemiological, clinical, and psychosocial studies on the benefits and risks of moderate drinking. These areas include, but are not limited to, coronary artery disease, hypertension, stroke, osteoporosis, breast cancer, interaction with various medications, risk/benefit analyses, and psychosocial issues.
- ***Neuroscience and behavior***. Studies of how alcohol’s effects on the central nervous system contribute to the development of intoxication, tolerance, and dependence.
- ***Prevention***. Research aimed at developing effective measures to reduce alcohol-related problems, including studies of alcohol-related intentional and unintentional injury, alcohol-related violence, alcohol in the workplace, drinking and driving deterrence, and the relationship between alcohol availability and alcohol-related problems.
- ***Treatment***. Research on improving diagnostic criteria and controlled studies to evaluate existing treatments and the impact of new treatment techniques, such as behavioral therapies.

**Intramural Research**

Scientists in the IRP focus on research opportunities that allow intensive, long-term commitment as well as the flexibility to adjust research priorities in response to new findings. Because clinical and laboratory studies occur side by side, new findings from basic research may be transferred readily for appropriate testing and application, and clinical hypotheses may, in turn, be posited to lab scientists.

Major IRP areas of study include the following: identification and assessment of genetic and environmental risk factors for the development of alcoholism; the effects of alcohol on the central nervous system, including how alcohol modifies brain activity and behavior; metabolic and biochemical effects of alcohol on various organs and systems of the body; noninvasive imaging of the brain’s structure and activity as it relates to alcohol use; development of animal models of alcoholism; the diagnosis, prevention, and treatment of alcoholism and associated disorders; and alcohol’s effects on a cellular level, including research on the proteins that compose membrane receptors and ion channels. Other studies on the expression of genes coding for these important proteins are yielding intriguing insights into the basic mechanisms of alcohol’s action. When combined with studies on region-specific effects of alcohol on the release of chemical messengers in the brain (i.e., neurotransmitters), these investigations will shed light on how alcohol produces reward, dependence, tolerance, and brain damage. Behavioral studies that primarily use mice, rats, and monkeys, combined with molecular genetics and behavioral manipulations during development, examine important protective and risk factors for alcohol abuse and dependence.

The IRP offers training opportunities, including clinical and laboratory programs oriented specifically to physicians; postdoctoral fellowships; and fellowships for students of medicine, social work, psychology, and other health professions. NIAAA encourages future scientists by providing training opportunities for high school, college, and graduate students.

## Applying for a Grant

Applications for NIAAA research grants are reviewed in a two-step process. First, an initial review group (i.e., the Study Section) comprised primarily of non-Federal experts in the applicant’s area of research evaluates the scientific merit of the application. Then, the National Advisory Council on Alcohol Abuse and Alcoholism (comprising scientific, professional, and public-sector leaders) reviews the Study Section’s recommendations and, as appropriate, may comment on the relevance and funding priority of the proposed project. Senior NIAAA staff make final determinations based on scientific merit, funding availability, and the Institute’s overall research objectives and program balance.

## Inquiries Are Welcome

For general information about alcohol abuse and alcoholism and NIAAA programs and activities, call or write: **Scientific Communications Branch**, 6000 Executive Blvd., Suite 409, Bethesda, MD 20892–7003, (301) 443–3860. Information also can be accessed via NIAAA’s World-Wide Web site at http://www.niaaa.nih.gov

NIAAA scientists and administrators are available to discuss research ideas and eligibility requirements for each grant or training opportunity. For additional information on specific research areas, contact the appropriate research division:

- **Division of Basic Research**, NIAAA, 6000 Executive Blvd., Suite 402, Bethesda, MD 20892–7003, (301) 443–2530.
- **Division of Biometry and Epidemiology**, NIAAA, 6000 Executive Blvd., Suite 514, Bethesda, MD 20892–7003, (301) 443–2193.
- **Division of Clinical and Prevention Research**, NIAAA, 6000 Executive Blvd., Suite 505, Bethesda, MD 20892–7003, (301) 443–1206.
- **Division of Intramural Clinical and Biological Research**, NIAAA, NIH Building 10, Room 3C103, 10 Center Drive MSC 1256, Bethesda, MD 20892–1256, (301) 496–8996 (Research training only).

**Major Types of Grants and Awards**

For a list of research announcements or for copies of individual announcements in a particular area, call or write: **National Clearinghouse for Alcohol and Drug Information**, P.O. Box 2345, Rockville, MD 20897–2345, (800) 729–6686. You may also access information concerning grant announcements from NIAAA or NIH World-Wide Web sites at http://www.niaaa.nih.gov (NIAAA) or http://www.nih.gov (NIH).

**Regular Research Grants (R01, R29):** Up to 5 years of support for basic or applied research on discrete projects.

**Exploratory Developmental Grants (R21):** One or two years of support for investigators building a capability to do alcohol research or conduct pilot studies leading to improved treatment research or to clinical trials.

**Small Grants (R03):** Two-year nonrenewable grants for exploratory or pilot studies, for developing a new technique or method, or for experts in other fields making a transition to alcohol research.

**Small Business Innovation Research Grants (R43 and R44):** Support for projects that seek to establish the technical merit and feasibility of ideas that may lead to a commercial project or service and for subsequent development of the product or service.

**Institutional Research Training Grants (T32):** Available to academic and medical institutions for training programs and for selecting pre- and postdoctoral students for up to 5 years of supervised research training in an alcohol-related specialty.

**Conference Grants (R13):** Support for conferences to exchange information or views or to explore or clarify a defined subject, problem, or area of knowledge.

**Career Development Awards:** Support for scientists and clinicians from health-related disciplines. The following categories of awards are available: ***Mentored Scientist Development Award* (K01)***—*Beginning scientists with previous research training and a doctoral degree in a science discipline who need further mentored research experience. ***Independent Scientist Award* (K02)***—*Fully trained scientists capable of designing and conducting independent research but requiring additional experience and skills to reach their full potential. ***Academic Career Award* (K07)***—*Faculty who have a clinical or research doctoral degree with potential to develop academic expertise in clinical alcohol research. ***Mentored Clinical Scientist Development Award* (K08)***—*Beginning scientists, trained primarily as clinicians, with a doctoral degree in a health field who require additional supervised research experience.

**Fellowships:** Support for future career development in alcohol research. Support is available for students enrolled in an established M.D./Ph.D. program **(F30)**; graduate students in a doctoral degree program in biomedical, biobehavioral, sociocultural, and other disciplines relevant to basic and applied research in alcohol abuse and alcoholism **(F31)**; students from minority groups underrepresented in biomedical and behavioral science; students with disabilities who are enrolled in a doctoral research degree program, a combined M.D./Ph.D. program, or another combined professional doctorate/research Ph.D. program **(F31-Special)**; persons with a doctoral degree who wish to obtain specialized training in research on the etiology, description, diagnosis, treatment, and prevention of alcoholism and the adverse consequences of alcohol abuse **(F32)**; and experienced scientists who wish to acquire training for major change in their research career or to enhance their scientific background through obtaining new capabilities to conduct alcohol-related research **(F33)**.

